# Acupressure versus NSAID for relief of orthodontic pain: A randomized controlled clinical trial

**DOI:** 10.1007/s00056-023-00476-0

**Published:** 2023-07-03

**Authors:** Moataz Elshehaby, Marwa Ali Tawfik, Mona A. Montasser

**Affiliations:** 1 Department of Orthodontic, Faculty of Oral and Dental Medicine Dentistry, Delta University for Science and Technology, Gamasa, Egypt; 2 Blanca Dental Clinics, Mansoura, Egypt; 3 Department of Orthodontic, Faculty of Dentistry, Mansoura University, Mansoura, Egypt

**Keywords:** Nonsteroidal anti-inflammatory agents, Tooth separator, Orthodontic appliances, Analgesics, Randomized controlled trial, Nichtsteroidale Antiphlogistika, Zahnseparator, Kieferorthopädische Apparaturen, Analgetika, Randomisierte kontrollierte Studie

## Abstract

**Aim:**

To compare ibuprofen and acupressure for pain relief after insertion of elastomeric orthodontic separators.

**Materials and methods:**

A randomized control clinical trial was conducted in an orthodontic clinic. A total of 75 orthodontic patients aged 12–16 years participating in the study were randomly allocated to receive either 400 mg of oral ibuprofen, applying acupressure therapy, or no pain-relief approach. Pain scores were recorded on visual analog scales (10 cm) over a week at different times (4, 10, 18, 24 h, and 1 week). The margin of equivalence was defined as 10 mm.

**Results:**

For all timepoints, the control group recorded the highest pain. For the ibuprofen and acupressure group, after 4 h, 18 h, and 1 week, no significant difference was noted. However, after 10 h, no significant difference in pain between the control and acupressure groups was noted and the ibuprofen group showed significantly lower pain. In the acupressure group, the highest pain was noted at 10 h. After this timepoint, pain progressively decreased with time and the lowest pain was noted after 1 week. In the control and ibuprofen groups, the highest pain was after 4 h, and then progressively decreased with time and the lowest pain was noted after 1 week.

**Conclusions:**

There was no significant difference in pain perception between participants using ibuprofen or acupressure and both groups recorded significantly lower pain than the control group at most of the observed timepoints. Results support the analgesic effect of the acupressure approach.

## Abstract (de)

**Zielsetzung:**

Vergleich von Ibuprofen und Akupressur zur Schmerzlinderung während des Einsetzens von kieferorthopädischen Elastomer-Separatoren.

**Materialien und Methoden:**

Eine randomisierte kontrollierte klinische Studie wurde in einer kieferorthopädischen Klinik durchgeführt. Insgesamt 75 kieferorthopädische Patienten im Alter von 12-16 Jahren, die an der Studie teilnahmen, wurden randomisiert einer Gruppe zugeteilt: 400 mg Ibuprofen oral, eine Akupressurtherapie oder keine schmerzlindernde Maßnahme. Die Schmerzwerte wurden auf visuellen Analogskalen (10 cm) über eine Woche zu verschiedenen Zeitpunkten (4, 10, 18, 24 Stunden und 1 Woche) aufgezeichnet. Die Äquivalenzgrenze wurde mit 10 mm definiert.

**Ergebnisse:**

Zu allen Zeitpunkten verzeichnete die Kontrollgruppe die stärksten Schmerzen. Nach 4 Stunden, 18 Stunden und einer Woche wurde in der Ibuprofen- und Akupressurgruppe kein signifikanter Unterschied festgestellt. Nach 10 Stunden wurde jedoch kein signifikanter Unterschied zwischen der Kontroll- und der Akupressurgruppe festgestellt, während die Patienten in der Ibuprofengruppe deutlich weniger Schmerzen hatten. Bei den Patienten der Akupressurgruppe waren die Schmerzen nach 10 Stunden am stärksten. Nach diesem Zeitpunkt nahmen die Schmerzen allmählich ab, und der geringste Schmerz wurde nach einer Woche festgestellt. In der Kontrollgruppe und der Ibuprofen-Gruppe war der Schmerz nach 4 Stunden am stärksten, nahm dann mit der Zeit ab und war nach einer Woche am geringsten.

**Schlussfolgerungen:**

Es gab keinen signifikanten Unterschied in der Schmerzwahrnehmung zwischen den Teilnehmern, die Ibuprofen oder Akupressur erhielten, und beide Gruppen verzeichneten zu den meisten der beobachteten Zeitpunkte deutlich geringere Schmerzen als die Kontrollgruppe. Die Ergebnisse unterstützen die schmerzlindernde Wirkung der Akupressur.

## Introduction

Almost all orthodontic patients experience some kind of pain at some point during their treatment. The prevalence of pain was found to range from 70–95%. It was considered one of the most common negative side effects of orthodontic treatment and a major concern for patients and clinicians as it could inversely affect the patients’ compliance or may dissuade them from continuing treatment [1, 2].

Pain could be elicited by many aspects of treatment: insertion of separators, use of leveling archwires or later on of heavy rectangular archwires, functional appliances, diverse active auxiliary components, or at the end by the debonding process [3–5]. From previous studies, it seems that all these measures were associated with pain [6–8].

The most common method to control pain is the use of analgesics and anti-inflammatory drugs [9, 10], which, however, may interfere with the rate of tooth movement [11].

Acupressure—sometimes called acupuncture without needles—is a nonpharmacological treatment method used in traditional Chinese medicine. It is a mechanical method of pain control that has been investigated in different medical and dental conditions [12–14]. The mechanism is to apply gentle manual pressure to specific trigger points on the body to relieve pain. These trigger points called acupressure points or acupoints are thought to work by enhancing normal blood flow or by stimulating the release of serotonin and endorphins responsible for counteracting the sensation of pain [13–16].

The goal of delivering painless orthodontic treatment has motivated research to examine new methods for pain relief. If effective in reducing orthodontic pain, acupressure would be a noninvasive, safe method that could be repeatedly practiced by children and adults as long as it is applied correctly. Therefore, this research aimed to compare the nonpharmacological intervention acupressure and a pharmacological intervention with the nonsteroidal anti-inflammatory drug (NSAID) ibuprofen (400 mg; Kahira Pharm. & Chem. Ind. Co., under license from Abbott Laboratories, Shobra, El Sahel, Egypt) with a control group on pain experienced by patients during the period of orthodontic teeth separation using elastomeric rings (Dentsply Raintree Essix, Sarasota, FL, USA).

## Materials and methods

### Study design, setting, and sample size calculation

A single-centered three parallel arms, longitudinal, prospective, randomized controlled clinical trial was conducted to reveal the effects of acupressure or the NSAID ibuprofen for relief of pain from orthodontic elastomeric separators.

For assessing the power of the study, G*Power [17] software (Heinrich-Heine-Universität Düsseldorf, Germany) was used to find the accepting type I statistical error of 5% and apply 2‑tailed statistical tests. Based on Hsieh et al. [18], the sample size was 15 participants per trial group. To avoid and counteract possible dropouts of participants for any reason, the group size was increased to 25 participants per group [19]. Thus, a total of 75 patients were needed for the study to have a group ratio of 1:1:1 (drug intervention 25, acupressure 25, and controls 25). Nine participants were dropped from the trial, whereby 5 participants did not practice the acupressure approach correctly, 2 did not show up again, and the other 2 forgot to complete the sheets.

### Eligibility criteria

Age ranged from 12–25 years. Furthermore, they had to present with healthy gingival tissue, no allergy to any analgesics, no history of any asthmatic steroid medication, or any other systemic diseases related to the kidney, liver, or heart.

### Exclusion criteria

Patients were not considered for the trial if there was previous orthodontic treatment, recent use of analgesics, any contraindications to NSAIDs, previous acupressure experience, inflamed gingival tissue, pregnancy, spacing between teeth, interproximal caries, or retained deciduous teeth.

### Participants

A total of 101 patients from the Department of Orthodontics, Faculty of Dentistry at Mansoura University who were enrolled for orthodontic treatment were screened to be included in the study. The planning and presentation of the study were guided by the Consolidated Standards of Reporting Trials (CONSORT) 2010 flow chart [20]. Approval for this randomized controlled trial was obtained from the ethical committee of the Faculty of Dentistry at Mansoura University (code: 05051217). Consent was obtained from the participants/parents before their recruitment in the trial, in a verbal and written manner. The examination and determination of eligibility criteria of each participant were performed by one examiner under the supervision of the trial coordinator.

**Fig. 1:**
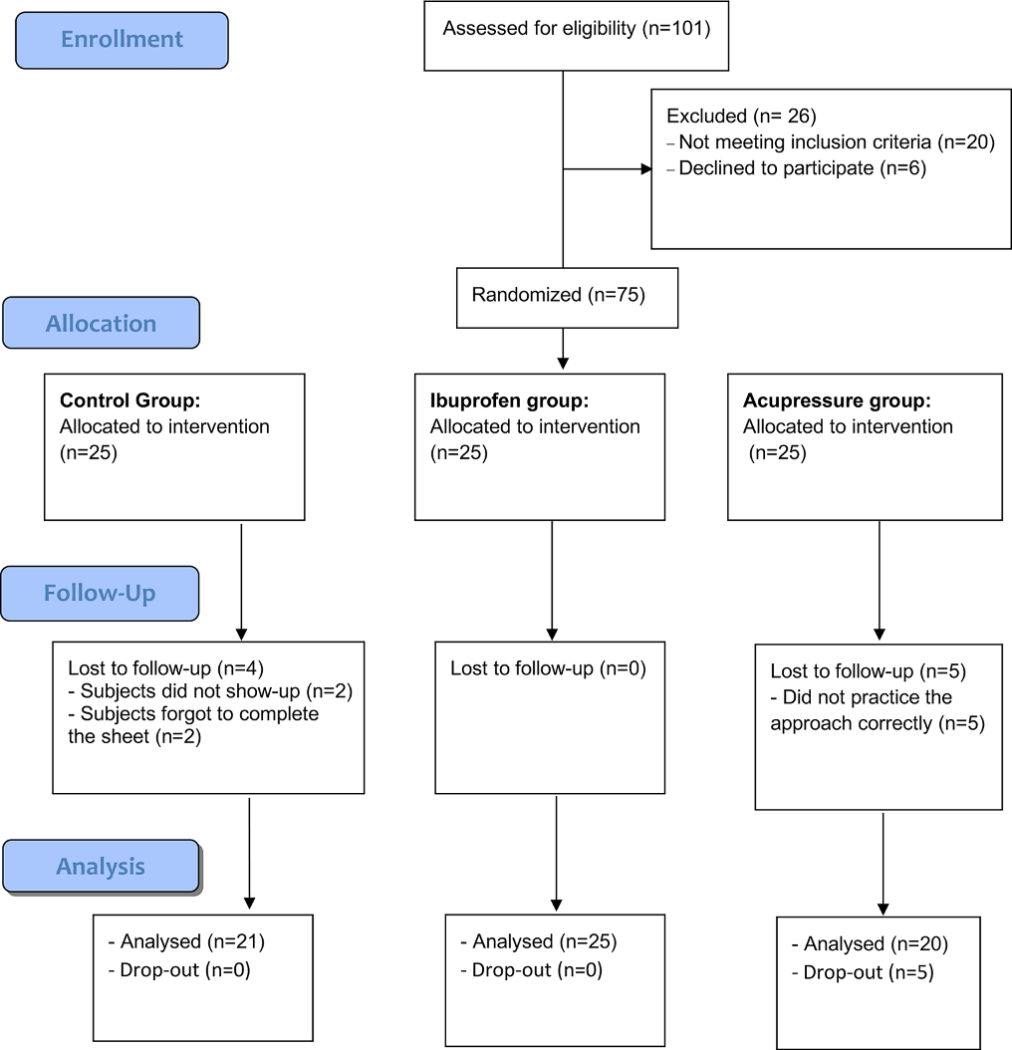
Consolidated Standards of Reporting Trials (CONSORT) flowchart CONSORT(Consolidated Standards of Reporting Trials)-Flussdiagramm

After applying the inclusion and exclusion criteria, 75 participants were randomly assigned to one of three groups to ensure that the size of the groups were similar. The participants of the same group practiced the same method of pain relief after the insertion of separators and until the next visit 7 days later. The CONSORT flow diagram is shown in Fig. 1.

At the start of orthodontic treatment, all participants received Duraseps elastic separators (Dentsply) in preparation to place bands on all four first molars. Separators were placed using placement pliers. The separator was stretched and guided in a slow controlled motion between the first molar and the neighboring tooth mesially and distally until the separator passed the contact area.

### Randomization and concealment

Central randomization was performed, and participant recruitment was done by phone.

The random allocation sequence was concealed in an envelope and held centrally. Participants were randomly assigned to three different groups and blinding was done using the SNOSE (sequentially numbered opaque sealed envelopes) technique. Each advice sheet was tightly sealed in one of the opaque envelopes identical in color, size, and weight prepared for that purpose. After shuffling, the participants’ names were replaced by codes on the envelopes and then stored with the trial coordinator, who was responsible for the randomization process and opening of the envelopes after finishing the trial.

### Intervention

The first intervention group was the NSAID group. The participants were instructed to read and apply the instructions of the advice sheet, dictating the use of ibuprofen (Kahira Pharm. & Chem. Ind. Co., under license from: Abbott Laboratories) 400 mg [1] with up to 4 doses in the first 24 h after the insertion of the separators for controlling the orthodontic pain (1 pill every 6 h). The toxicity of this dose is far below the established toxicity level, which is 3500 mg [21].

**Fig. 2:**
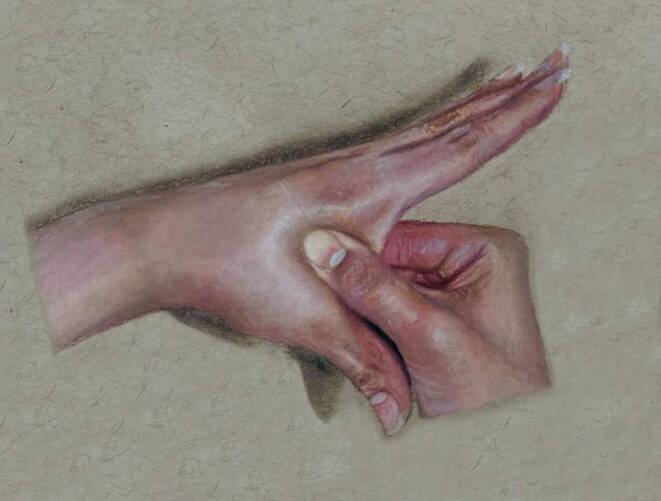
Acupressure application Anwendung der Akupressur

The second intervention group was the acupressure group. The participants received an advice sheet with instructions to practice acupressure by applying pressure to a defined acupressure point on the back of the hand to control orthodontic pain as often as needed for the first 24 h only after insertion of the separators. The point LI4 for facial pain control is located between the thumb and index digits of the hand at the center between the first and second metacarpal bones. The point was explained to the participants with the help of a drawing (Fig. 2).

The third group was the control group; no specific pain relief intervention following separator placement was performed.

All the participants were instructed that an extra dose of analgesic should not be needed; however, if extreme pain sensation was felt after doing the pain-relieving approach, either ibuprofen or acupressure, a supplementary dose of paracetamol could be taken 4 h after from the previous pain-relieving approach performed to avoid overlapping of the two analgesic effects, or a minimum of 4 h from the insertion of the separators for the control group to be sure that the pain had not reduced yet. All the advice sheets had a blank field to include the supplementary medication type, dose taken, and time. The participants taking the rescue dose were considered drop-out participants. All participants were guided to immediately call by phone or come to the orthodontic department in case any adverse reactions developed.

### Outcome

All the information needed for the participants was given to them using advice sheets written in Arabic. To ensure that the advice sheet instructions could be easily understood by the participants, a pilot study with 10 participants was carried out per group.

The participants were asked to log the intensity of the pain that they experienced after the insertion of the separators using a pain diary and a visual analog scale (VAS) [22]. The pain diary covered the timepoints: 4 h after insertion, after eating (10 h), after sleeping (18 h), after 24 h, and after 7 days. The rationale for this schedule was based on the onset of the analgesic action and the drug half-life times plus the short onset of time for acupressure known from the previous studies.

**Fig. 3:**
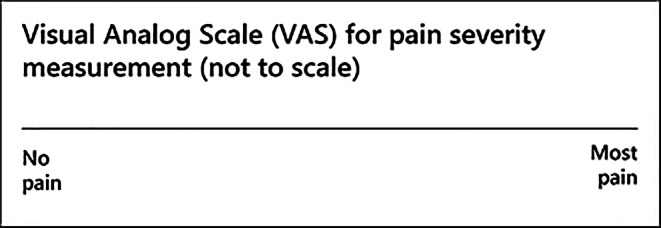
Visual analog scale Visuelle Analogskala

The length of the visual analog scale was 10 cm but without intermediate digits, to avoid choosing a random number by the participant and to give the participant the chance to correctly indicate how severe the pain was on a solid straight line [22]. The VAS score was determined by measuring in millimeters from the beginning left of the line to the vertical line that the participant marked on the horizontal straight line of the scale (Fig. 3).

After the advice sheet and the questionnaires were completed, the examiners made all measurements to the approximation of 0.5 mm with a ruler made of stainless steel. Inter- and intrareliability tests were performed to ensure that reliable results were obtained.

### Statistical analyses

Data were analyzed using SPSS® software version 22 (IBM, Armonk, NY, USA). Interpersonal and intrapersonal reliability were tested using Cronbach’s alpha with a correlation coefficient > 0.80 to assess the reliability of the data. The normality of data distribution was tested using the Shapiro–Wilk test. As the data were parametric and normally distributed, it was described as mean ± standard deviation [SD]. A graphic presentation of data was performed using clustered bars and error bar charts. The analysis of variance (ANOVA) test was used to compare VAS between measurement times with the group as an independent factor. The Bonferroni test was used for multiple comparisons if significant differences were detected. A probability value of less than 0.05 was set for statistical significance.

## Results

The results were considered reliable as the interpersonal and intrapersonal correlation coefficients were > 0.80.

All measurements were parametric and normally distributed (Shapiro–Wilk test, *p* value > 0.05).

**Fig. 4:**
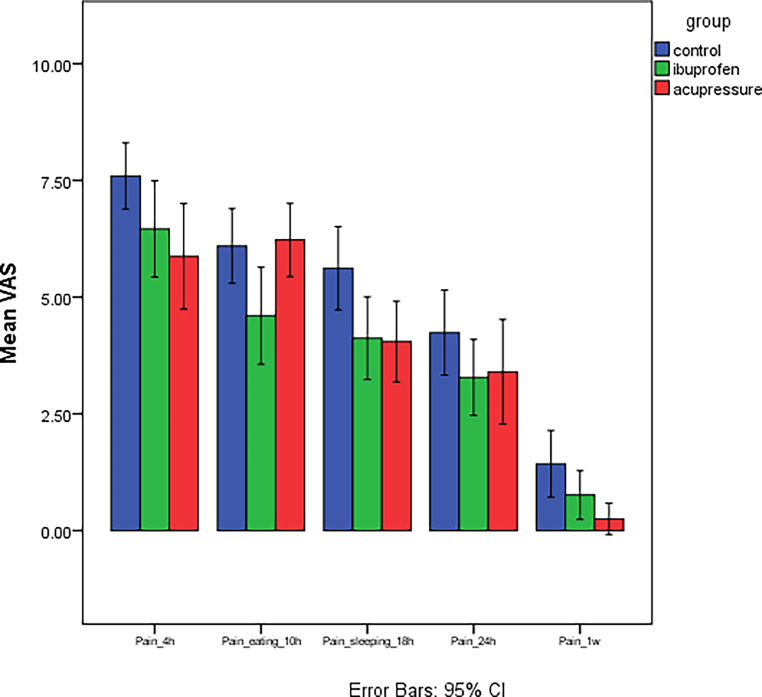
Mean visual analog scale (VAS) pain scores at the various observation times. *95% CI *95% confidence interval Durchschnittliche VAS(visuelle Analogskala)-Scores für Schmerz zu den unterschiedlichen Beobachtungszeitpunkten. *95% CI *95%-Konfidenzintervall

For all measurement times, the highest pain was recorded in the control group. For the ibuprofen and the acupressure groups, no significant difference in pain was noted after 4 h, 18 h, and 1 week and these two groups recorded significantly lower pain than the control group. However, after 10 h, no significant difference in pain between the control and acupressure groups was noted, while the ibuprofen group showed significantly lower pain than these two groups at this timepoint (Table 1 and Fig. 4). For the acupressure group, the highest pain was noted at 10 h, followed by 4 h (with no significant difference between these times); pain then progressively decreased with time at 18 h and 24 h (with no significant difference between these times). The lowest pain was noted after 1 week.

**Table 1:** Comparison of the mean visual analog scale pain levels for all groups at each observation time Vergleich der mittleren Schmerzwerte auf der visuellen Analogskala für alle Gruppen zu jedem Beobachtungszeitpunkt

	Control		Ibuprofen		Acupressure		*P* value
	Mean	SD	Mean	SD	Mean	SD	
Pain_4 h	7.6^a^	1.65	6.46^b^	2.49	5.88^b^	2.42	0.046*
Pain_eating_10 h	6.1^a^	1.76	4.60^b^	2.52	6.23^a^	1.68	0.015*
Pain_sleeping_18 h	5.62^a^	1.96	4.12^b^	2.15	4.05^b^	1.68	0.020*
Pain_24 h	4.24^a^	2.00	3.28^a^	1.97	3.40^a^	2.39	0.27
Pain_1 week	1.43^a^	1.57	0.76^b^	1.27	0.25^b^	0.72	0.013*

Different letters in the same row indicated a significant difference in means between the two groups (Bonferroni, *P is significant at < 0.05)

*SD* standard deviation

**Fig. 5:**
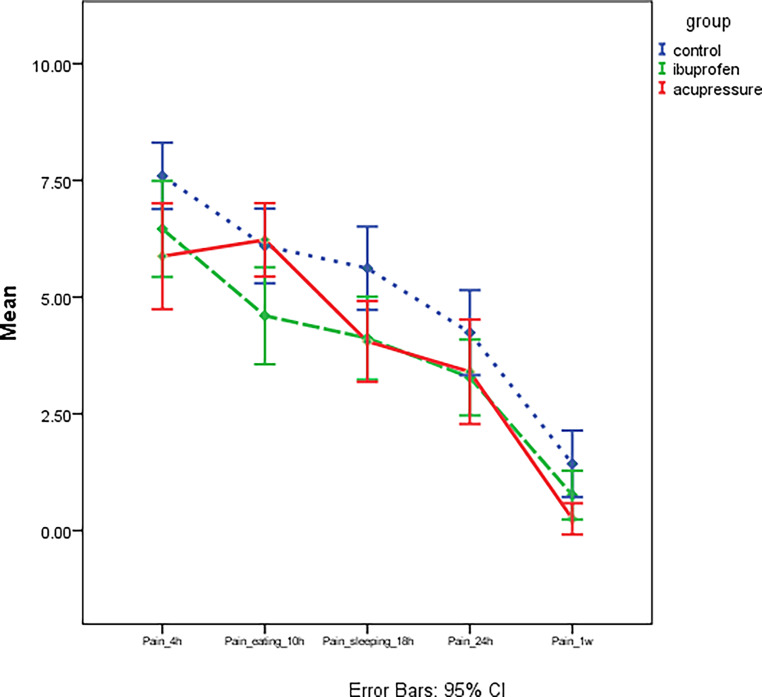
Comparison of mean visual analog scale (VAS) pain scores at various observation times for each group. *95% CI *95% confidence interval Vergleich der durchschnittlichen VAS(visuelle Analogskala)-Scores für Schmerz zu den unterschiedlichen Beobachtungszeitpunkten für jede Gruppe. *95% CI *95%-Konfidenzintervall

For the control and the ibuprofen groups, the highest pain was noted after 4 h, and then the pain progressively decreased over time at subsequent measurement times (at 10 h and 18 h there was no difference, while pain continued to decrease at 24 h) with the lowest pain being noted after 1 week. Comparisons between the observation times for each group are presented in Table 2 and Fig. 5.

**Table 2:** Comparison of mean visual analog scale (VAS) pain scores at the various observation times Vergleich der durchschnittlichen VAS(visuelle Analogskala)-Scores für Schmerz zu den unterschiedlichen Beobachtungszeitpunkten

	Control		Ibuprofen		Acupressure	
	Mean	SD	Mean	SD	Mean	SD
Pain_4 h	7.6^a^	1.65	6.46^b^	2.49	5.88^b^	2.42
Pain_eating_10 h	6.1^a^	1.76	4.60^b^	2.52	6.23^a^	1.68
Pain_sleeping_18 h	5.62^a^	1.96	4.12^b^	2.15	4.05^b^	1.68
Pain_24 h	4.24^a^	2.00	3.28^a^	1.97	3.40^a^	2.39
Pain_1 week	1.43^a^	1.57	0.76^b^	1.27	0.25^b^	0.72
*P* value	< 0.001*		< 0.001*		< 0.001*	

Different letters in the same column indicated a significant difference in means between the two observation times (Bonferroni, *P is significant at < 0.05)

*SD* standard deviation

## Discussion

Placement of elastic separators commonly results in pain [23]. Thus, controlling pain is important as it supports the success of orthodontic treatment. This study aimed to reveal whether acupressure is as effective as ibuprofen medication for relieving orthodontic pain after the insertion of separators.

Numerous previous studies on pain control showed no correlation between pain and patient’s sex, likewise in the present study, no sex correlation was found and the sexes were combined for the data analysis [24, 25].

Acupressure, a nonpharmacological pain-relieving approach, is becoming globally known and usable because this approach is seen as an effective, yet very conservative approach in comparison with the conventional use of pharmacological drugs. On the other hand, recently available evidence is largely inconclusive.

In the present study, a clear pattern was seen for the onset of pain in the three groups: the pain reached its highest level after 4 h and then started to decrease progressively. Ngan et al. [8] used a visual analog scale to evaluate the level of perception of discomfort by orthodontic patients after the insertion of separators and found a significant increase in pain after separator insertion at 4 h and 24 h.

The course of decline in pain perception is considered to be an indication of a decrease of pain by time and/or the loss of separator elasticity.

In the control group and also in the ibuprofen group, there was no significant difference in pain perception at 10 h and 18 h, even though the pain level in the ibuprofen group was less, indicating superior analgesic activity compared with the control group [26, 27].

In the acupressure group, there was no significant difference in pain perception at 4 h and 10 h and also at 18 h and 24 h, indicating a significant decrease in pain perception at the 10 h timepoint. This could be attributed to the patients developing increasing skills in performing the acupressure technique and becoming confident with its effectiveness and, thus, performing it more effectively and regularly.

In the present study, the control group recorded the highest pain level among the three groups at all timepoints except after 10 h or during eating where the pain perception was not different between this group and the acupressure group. The acupressure group showed a nonsignificantly higher pain perception than the control group. This may partly explain the dropouts in the current study.

Drop-outs in the control group mainly occurred because of intolerable pain sensation or the participants’ decision to stop orthodontic treatment from the beginning. The acupressure dropouts occurred when a participant did not fulfill the advice sheet properly (was not able to practice the maneuver correctly or was not compliant with doing the acupressure at all), so was excluded from the study. It is highly recommended to test the participant’s understanding of the acupressure technique and application before advising the participant to use it for relieving orthodontic pain.

Acupressure and ibuprofen groups recorded significantly lower pain perception than the control group at 4 h, 18 h, and 1 week because the participants were instructed to perform acupressure at any time and an indefinite number of times. Thus, participants were under the umbrella of pain-relieving approaches all day long during the first 24 h. However, after 10 h or during eating, the ibuprofen group showed significantly lower pain perception than the other two groups. In the acupressure group, this could be attributed to the nature of the acupressure technique requiring frequent application. Patients at this early time may have not yet have found the appropriate rate for them to do the acupressure. Between the ibuprofen and the acupressure groups after 4 h, 18 h, 24 h, and 1 week, no significant difference in pain perception was noted. This could be considered a promising result confirming the effectiveness of the acupressure approach for orthodontic pain relief with a similar effect to that of ibuprofen especially at the time of the peak of pain that occurred 4 h after insertion of the separators.

In the present study design, there were 4 doses of ibuprofen, once every 6 h, unlike other studies where participants received medication after 3 or 4 h after insertion of the separators.

In summary, acupressure could be an important alternative analgesic for pain induced by orthodontic elastic separators. Further studies are recommended to study its effect on orthodontic pain induced by other procedures and to find out the appropriate rate of application.

## Conclusions

- Orthodontic pain resulting from the insertion of elastomeric separators reached a peak after 4 h, then decreased reaching the minimum at the end of the first week.
- There was no significant difference in pain perception between participants using ibuprofen or acupressure at the time of the peak of pain (after 4 h), after 18 h, after 24 h, and after 1 week.
- Both the ibuprofen and the acupressure groups recorded significantly lower pain than the control group at most of the timepoints which supports the analgesic effect of the acupressure approach.
- Further studies evaluating the long-term effect of acupressure and its analgesic effect for other orthodontic treatment procedures are recommended.

## References

[1] Scheurer PA, Firestone AR, Bürgin WB (1996) Perception of pain as a result of orthodontic treatment with fixed appliances. Eur J Orthod 18(1):349–357 8921656 10.1093/ejo/18.4.349

[2] Oliver R, Knapman Y (1985) Attitudes to orthodontic treatment. Br J Orthod 12(4):179–188 3863673 10.1179/bjo.12.4.179

[3] Jones M, Chan C (1992) The pain and discomfort experienced during orthodntic treatment: A randomized controlled clinical trial of two intial aligning arch wires. Am J Orthod Dentofacial Orthop 102(4):373–381 1456222 10.1016/0889-5406(92)70054-e

[4] Giannopoulou C, Dudic A, Kiliaridis S (2006) Pain discomfort and crevicular fluid changes induced by orthodontic elastic separators in children. J Pain 7(5):367–376 16632326 10.1016/j.jpain.2005.12.008

[5] Krishnan V (2007) Orthodontic pain: from causes to management—a review. Eur J Orthod 29(2):170–179 17488999 10.1093/ejo/cjl081

[6] Jones M (1984) An investigation into the initial discomfort caused by placement of an archwire. Eur J Orthod 6(1):48–54 6583064 10.1093/ejo/6.1.48

[7] Needleman HL, Hoang C, Allred E, Hertzberg J, Berde C (2000) Reports of pain by children undergoing rapid palatal expansion. Pediatr Dent 22(3):221–226 10846733

[8] Ngan P, Kess B, Wilson S (1989) Perception of discomfort by patients undergoing orthodontic treatment. Am J Orthod Dentofacial Orthop 96(1):47–53 2750720 10.1016/0889-5406(89)90228-x

[9] Angelopoulou M, Vlachou V, Halazonetis D (2012) Pharmacological management of pain during orthodontic treatment: a meta-analysis. Orthod Craniofac Res 15(2):71–83 22515183 10.1111/j.1601-6343.2012.01542.x

[10] Gupta M, Kandula S, Laxmikanth SM, Vyavahare SS, Reddy SB, Ramachandra CS (2014) Controlling pain during orthodontic fixed appliance therapy with non-steroidal anti-inflammatory drugs (NSAID): a randomized, double-blinded, placebo-controlled study. J Orofac Orthop 75(6):471–476 25355194 10.1007/s00056-014-0243-7

[11] Karthi M, Anbuslevan GJ, Senthilkumar KP, Tamizharsi S, Raja S, Prabhakar K (2012) NSAIDs in orthodontic tooth movement. J Pharm Bioallied Sci 4(Suppl 2):S304 23066276 10.4103/0975-7406.100280 PMC3467920

[12] Lee EJ, Frazier SK (2011) The efficacy of acupressure for symptom management: a systematic review. J Pain Symptom Manage 42(4):589–603 21531533 10.1016/j.jpainsymman.2011.01.007 PMC3154967

[13] Chen Y‑W, Wang H‑H (2014) The effectiveness of acupressure on relieving pain: a systematic review. Pain Manag Nurs 15(2):539–550 23415783 10.1016/j.pmn.2012.12.005

[14] Mangal B, Sugandhi A, Kumathalli KI, Sridhar R (2012) Alternative medicine in periodontal therapy—a review. J Acupunct Meridian Stud 5(2):51–56 22483182 10.1016/j.jams.2012.01.001

[15] Organization WH (1991) A proposed standard international acupuncture nomenclature: report of a WHO scientific group. World Health Organization

[16] Tsay S‑L (2004) Acupressure and fatigue in patients with end-stage renal disease—a randomized controlled trial. Int J Nurs Stud 41(1):99–106 14670399 10.1016/s0020-7489(03)00079-8

[17] Faul F, Erdfelder E, Lang A‑G, Buchner A (2007) G* Power 3: A flexible statistical power analysis program for the social, behavioral, and biomedical sciences. Behav Res Methods 39(2):175–191 17695343 10.3758/bf03193146

[18] Hsieh LL‑C, Liou H‑H, Lee L‑H, Chen TH‑H, Yen AM‑F (2010) Effect of acupressure and trigger points in treating headache: a randomized controlled trial. Am J Chin Med 38(01):1–14 20128040 10.1142/S0192415X10007634

[19] Guo Y, Pandis N (2015) Sample-size calculation for repeated-measures and longitudinal studies. Am J Orthod Dentofac Orthop 147(1):146–149 10.1016/j.ajodo.2014.10.009 25533082

[20] Schulz KF, Altman DG, Moher D (2010) CONSORT 2010 statement: updated guidelines for reporting parallel group randomized trials. Ann Intern Med 152(11):726–732 20335313 10.7326/0003-4819-152-11-201006010-00232

[21] Davies NM (1998) Clinical pharmacokinetics of ibuprofen. Clin Pharmacokinet 34(2):101–154 9515184 10.2165/00003088-199834020-00002

[22] Hawker GA, Mian S, Kendzerska T, French M (2011) Measures of adult pain: Visual analog scale for pain (vas pain), numeric rating scale for pain (nrs pain), mcgill pain questionnaire (mpq), short-form mcgill pain questionnaire (sf-mpq), chronic pain grade scale (cpgs), short form-36 bodily pain scale (sf-36 bps), and measure of intermittent and constant osteoarthritis pain (icoap). Arthritis Care Res 63:S11 10.1002/acr.20543 22588748

[23] Bird SE, Williams K, Kula K (2007) Preoperative acetaminophen vs ibuprofen for control of pain after orthodontic separator placement. Am J Orthod Dentofac Orthop 132(4):504–510 10.1016/j.ajodo.2006.11.019 17920504

[24] Dionne RA, Campbell RA, Cooper SA, Hall DL, Buckingham B (1983) Suppression of postoperative pain by preoperative administration of ibuprofen in comparison to placebo, acetaminophen, and acetaminophen plus codeine. J Clin Pharmacol 23(1):37–43 6341415 10.1002/j.1552-4604.1983.tb02702.x

[25] Erdinç EAM, Dinçer B (2004) Perception of pain during orthodontic treatment with fixed appliances. Eur J Orthod 26(1):79–85 14994886 10.1093/ejo/26.1.79

[26] Bernhardt MK, Southard KA, Batterson KD, Logan HL, Baker KA, Jakobsen JR (2001) The effect of preemptive and/or postoperative ibuprofen therapy for orthodontic pain. Am J Orthod Dentofac Orthop 120(1):20–27 10.1067/mod.2001.115616 11455373

[27] Polat O, Karaman AI, Durmus E (2005) Effects of preoperative ibuprofen and naproxen sodium on orthodontic pain. Angle Orthod 75(5):791–796 16279825 10.1043/0003-3219(2005)75[791:EOPIAN]2.0.CO;2

